# Supplementary material for: Electric vehicle batteries alone could satisfy short-term grid storage demand by as early as 2030
Source: Nat Commun. 2023 Jan 17;14:119. doi: 10.1038/s41467-022-35393-0 (PMC9845221; doi:10.1038/s41467-022-35393-0)
Supplement: Supplementary file 1 — Supplementary Information [file 41467_2022_35393_MOESM1_ESM.pdf]

Electric vehicle batteries alone could satisfy short-term grid storage demand by as early as 2030

Chengjian Xu[1]*, Paul Behrens[1], Paul Gasper[2], Kandler Smith[2], Mingming Hu[1], Arnold Tukker[1,3], Bernhard Steubing[1]

1. Institute of Environmental Sciences (CML), Leiden University, 2300, RA Leiden, The Netherlands
2. National Renewable Energy Lab, 15013 Denver West Pkwy, Golden, Colorado, U.S.A.
3. Netherlands Organisation for Applied Scientific Research TNO, 2595 DA Den Haag, Netherlands

* Corresponding Author: xuchegjian@gmail.com

Model overview

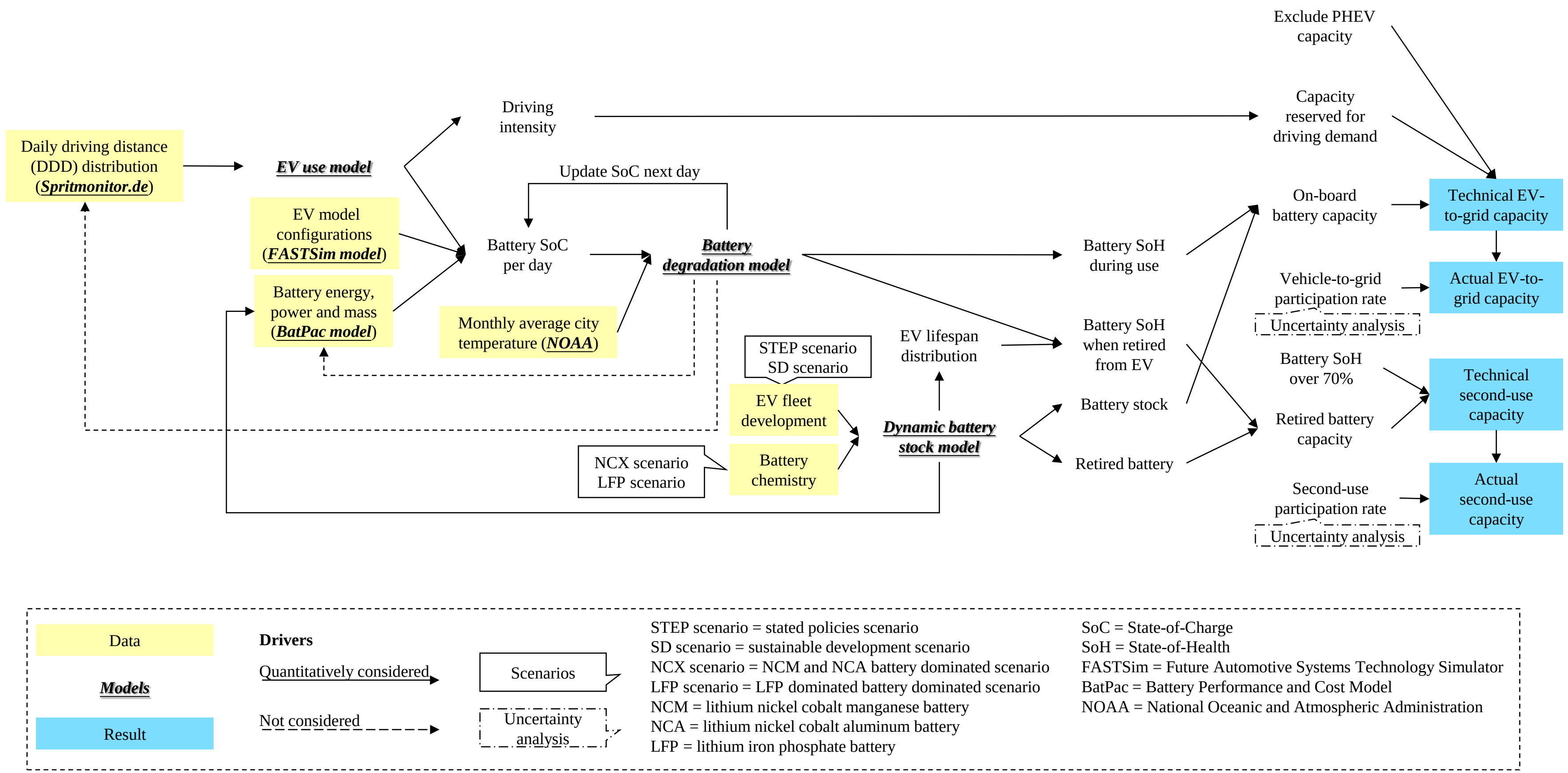

Supplementary Fig. 1: Model framework consisting of a dynamic battery stock model, a EV use model, and a battery degradation model.

Dynamic battery stock model

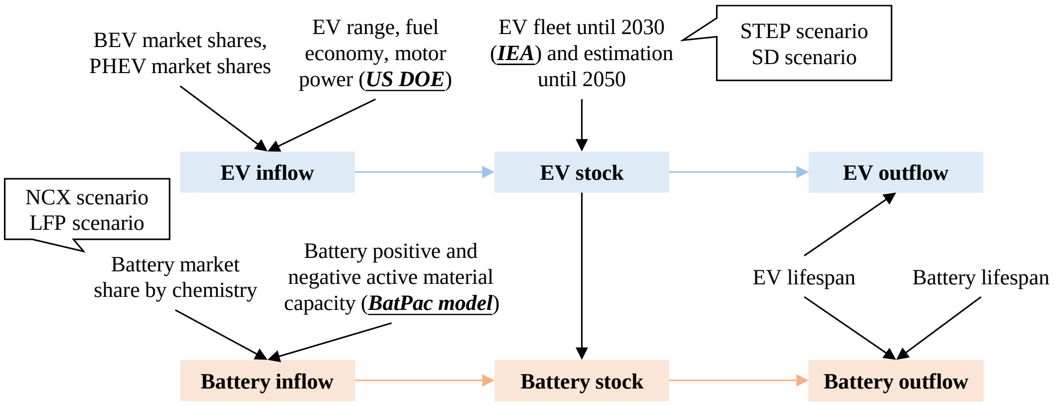

STEP scenario = stated policies scenario
SD scenario = sustainable development scenario
NCX scenario = NCM and NCA battery dominated scenario
LFP scenario = LFP dominated battery dominated scenario
NCM = lithium nickel cobalt manganese battery
NCA = lithium nickel cobalt aluminum battery
LFP = lithium iron phosphate battery

IEA = International Energy Agency
USDOE = U.S. Department of Energy
BatPac = Battery Performance and Cost Model

Supplementary Fig. 2: Dynamic battery stock model[1].

EV use model

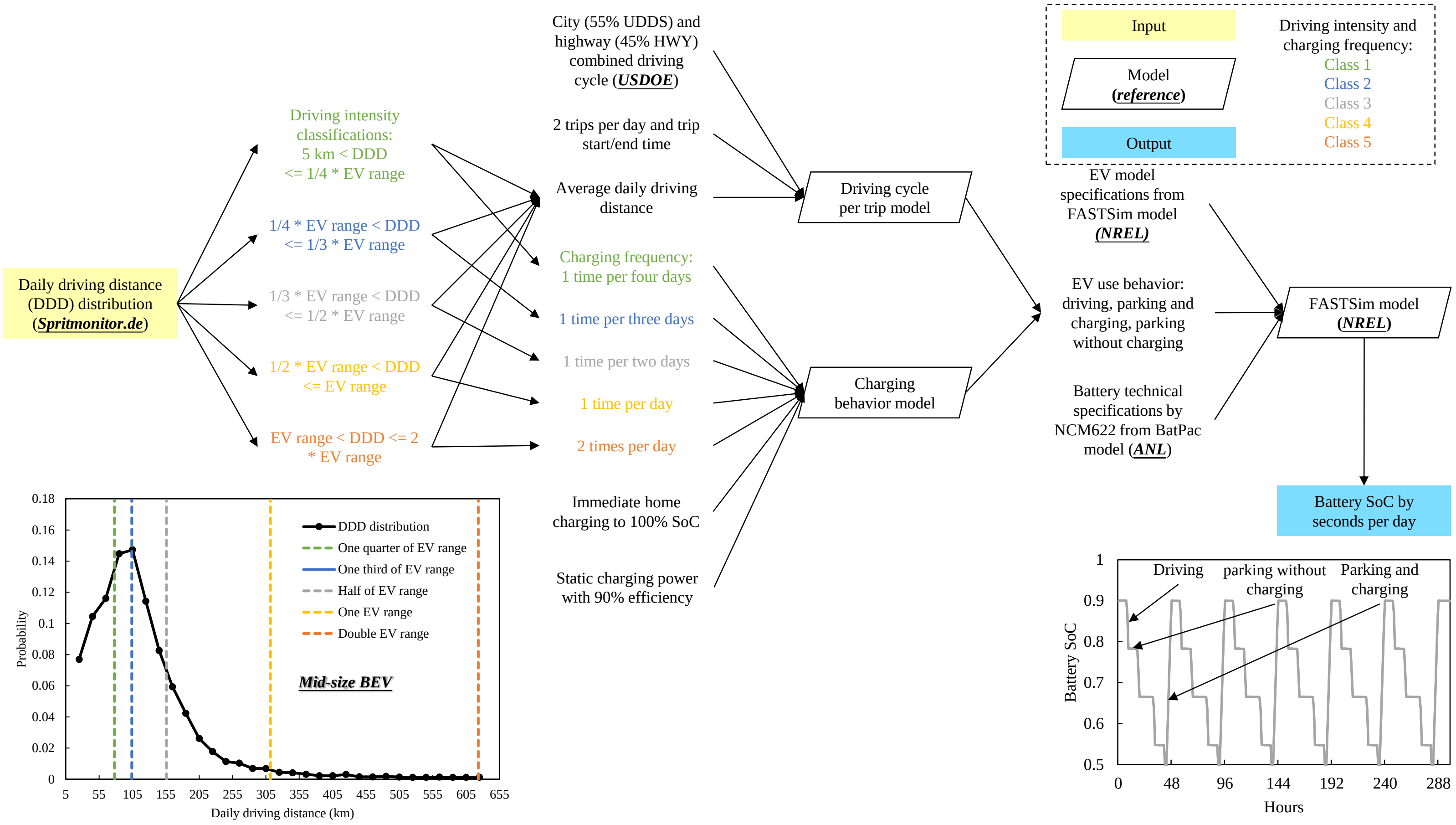

Supplementary Fig. 3: EV use model. NREL National Renewable Energy Laboratory. ANL Argonne National Laboratory.

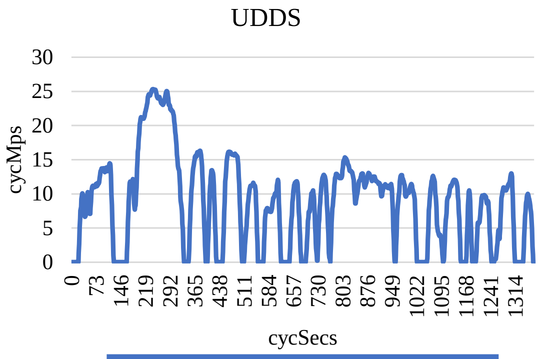

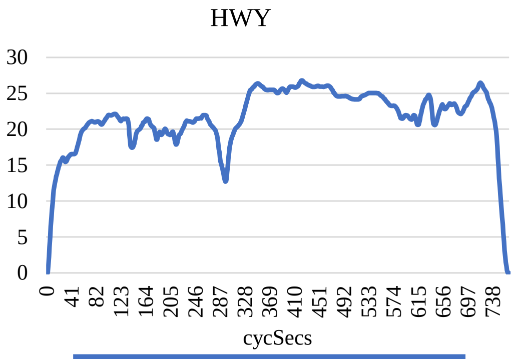

UDDS	
Cumkm	11.99043319
Average speed (m/s)	8.752141014
Time (s)	1369

HWY	
Cumkm	16.50681747
Average speed (m/s)	21.54937007
Time (s)	765

Average daily driving distance = x

Trip distance = 0.5*x

Required UDDS trip distance = 0.55*0.5*x

Required UDDS trip time = ROUND (0.55*0.5*x / UDDS average speed, 0)

Required multiples of UDDS = FLOOR (Required UDDS trip time / UDDS trip time, 1)

Required downsize factor UDDS = Round (UDDS trip time / MOD (Required UDDS trip time / UDDS trip time), 2)

Required HWY trip distance = 0.45*0.5*x

Required HWY trip time = ROUND (0.45*0.5*x / HWY average speed, 0)

Required multiples of HWY = FLOOR (Required HWY trip time / HWY trip time, 1)

Required downsize factor HWY = Round (HWY trip time / MOD (Required HWY trip time / HWY trip time), 2)

For UDDS and HWY, **first scale the resolution from 1s to 0.01 s**. new cycMps (for cycSecs in 0.00-0.49, 0.01s interval) = old cycMps (0); new cycMps (0.50-1.49) = old cycMps (1); new cycMps (1.50-2.49) = old cycMps (2) …

Downsized cycMps (0) = average (new cycMps (0, downsize factor));

Downsized cycMps (1) = average (new cycMps (downsize factor+0.01, 2*downsize factor+0.01)) …

Supplementary Fig. 4: EV use model where driving cycle is compiled on trip distance and standard UDDS and HWY driving cycle.

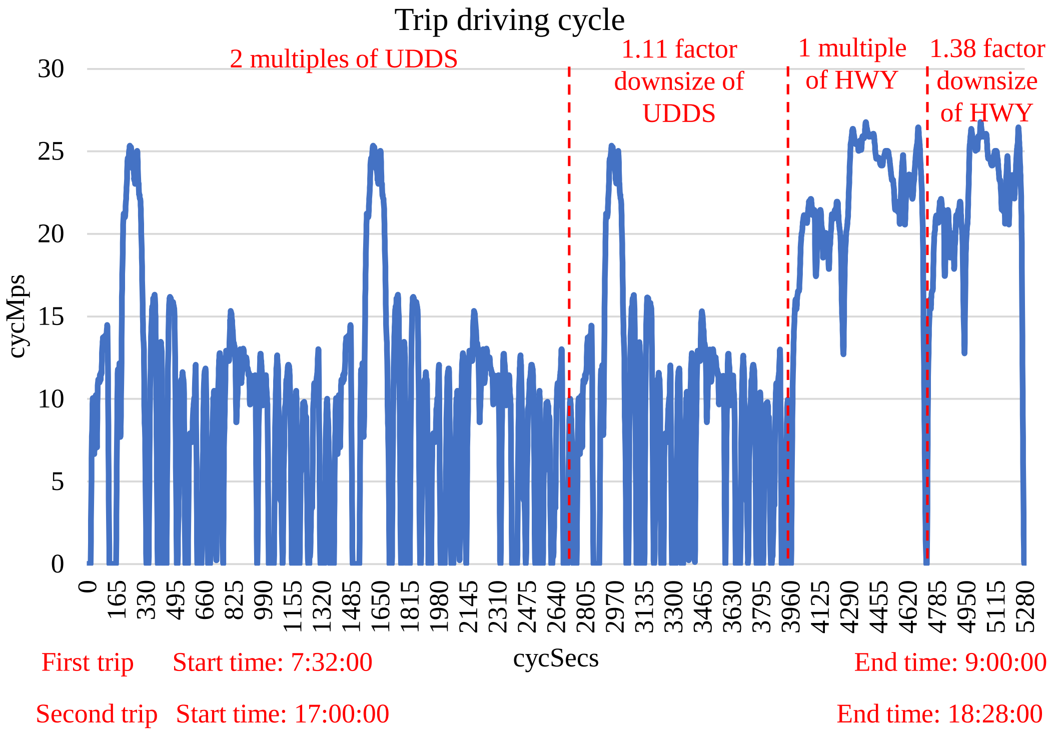

Supplementary Fig. 5: EV use model where a drive cycle example is compiled for a mid-size BEV when the daily driving distance is 126.3 km.

Battery degradation model

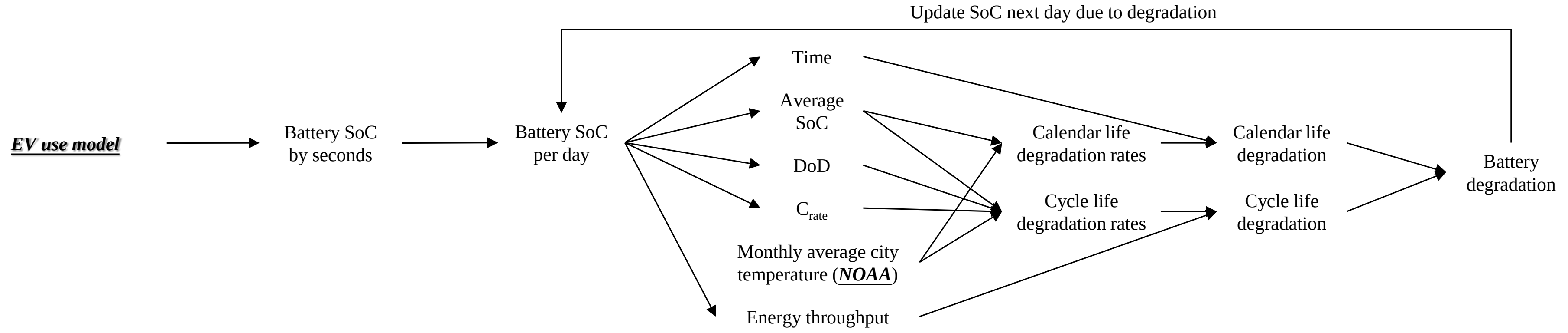

Supplementary Fig. 6: Battery degradation model.

Additional Figures and Tables

Supplementary Figures

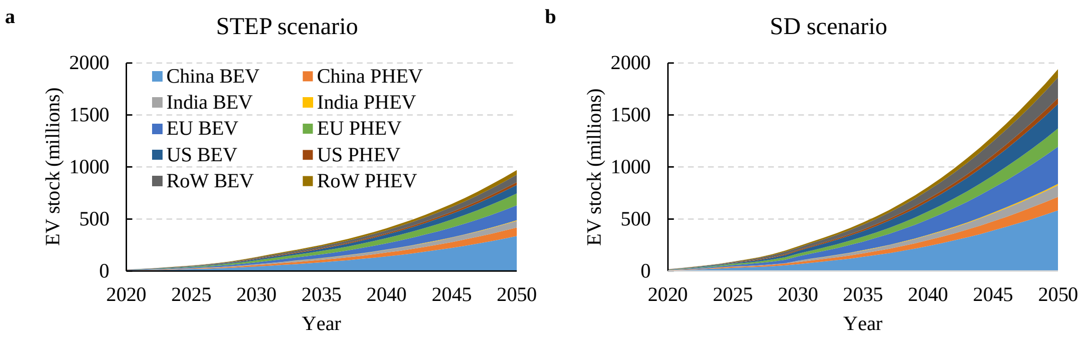

Supplementary Fig. 7: Global EV stock development projected until 2050 for STEP and SD fleet scenarios. a STEP scenario. **b** SD scenario. BEV battery electric vehicle, PHEV plug-in hybrid electric vehicle, STEP scenario the Stated Policies scenario, SD scenario Sustainable Development scenario.

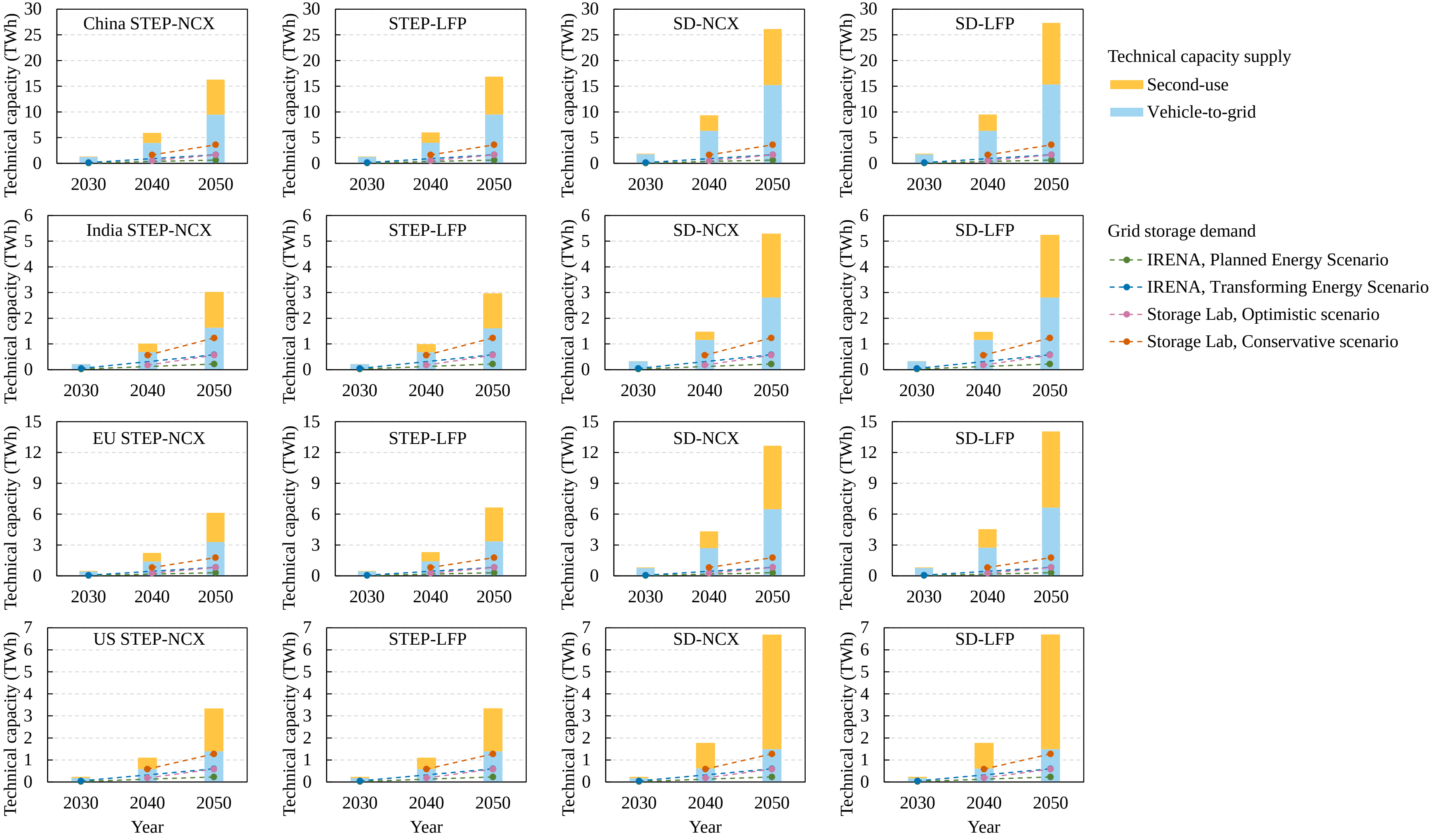
China STEP-NCX
STEP-LFP
SD-NCX
SD-LFP
India STEP-NCX
EU STEP-NCX
US STEP-NCX
Technical capacity (TWh)
Year
2030
2040
2050
Technical capacity supply
Second-use
Vehicle-to-grid
Grid storage demand
IRENA, Planned Energy Scenario
IRENA, Transforming Energy Scenario
Storage Lab, Optimistic scenario
Storage Lab, Conservative scenario

Supplementary Fig. 8: Total technical capacity from EV batteries and comparison to grid storage demand in countries and regions. The grid storage demand in countries/regions is estimated based on future peak power demand in countries/regions, where assuming a proportional relationship between grid storage demand and peak power demand for countries/regions is the same as global. Global peak power will increase to 6686 GW in 2030 and 10000 GW in 2050, derived from Storage Lab[2]. China's peak power will increase to 1258 GW in 2030 and 1881 GW in 2050. India's peak power will increase to 430 GW in 2030 and 643 GW in 2050. EU peak power will increase to 616 GW in 2030 and 922 GW in 2050. US peak power will increase to 445 GW in 2030 and 665 GW in 2050. Regional peak demand is from the IEA[3].

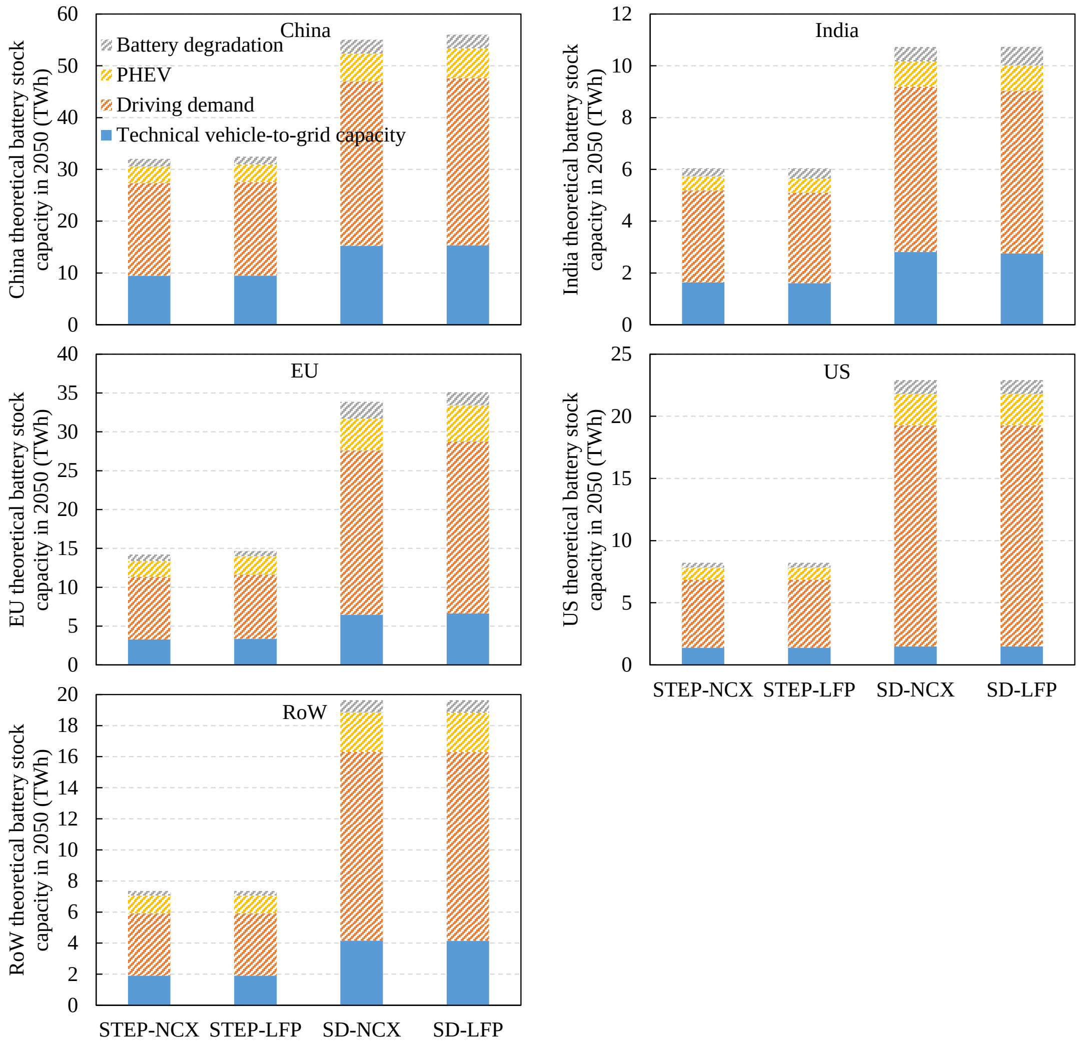

Supplementary Fig. 9: Available vehicle-to-grid capacity in 2050 by countries/regions. Hatched bars indicate the capacity limits due to key factors and blue bars the technical vehicle-to-grid capacity. It is found higher technical vehicle-to-grid capacity for LFP scenario compared to NCX scenario in China, EU, and US, while higher vehicle-to-grid capacity for the NCX scenario in India and RoW (Rest of World).

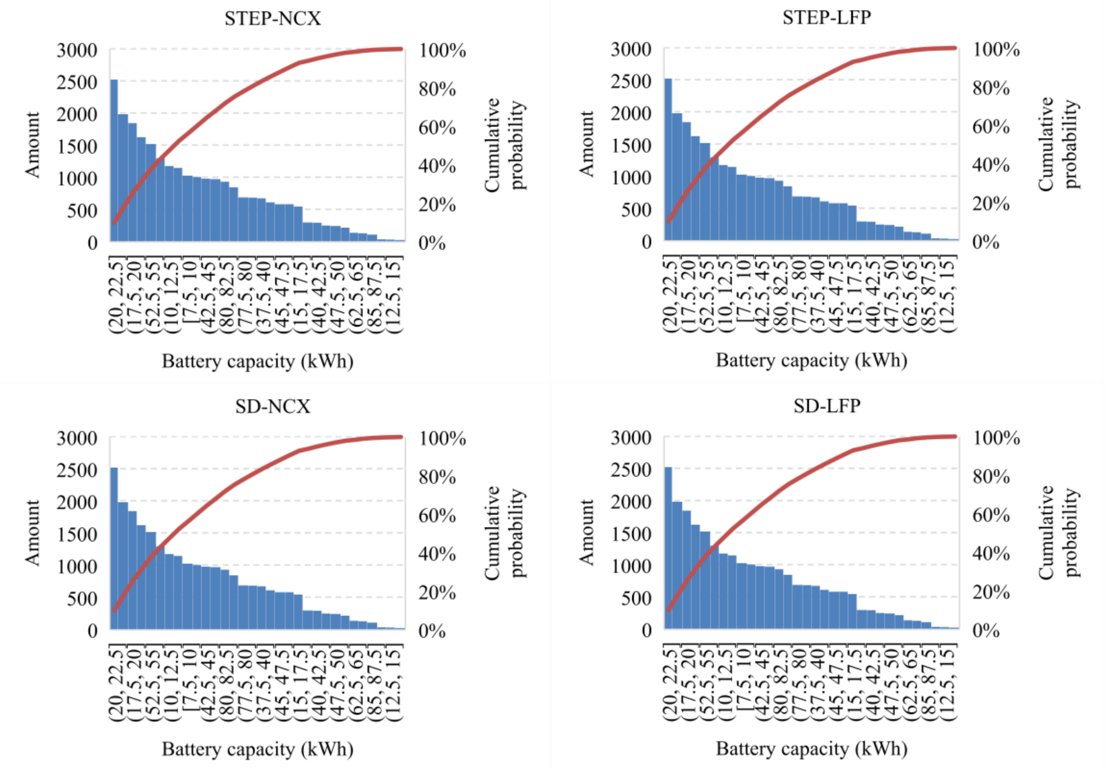

Supplementary Fig. 10: Battery capacity distribution for China battery stock by 2050.

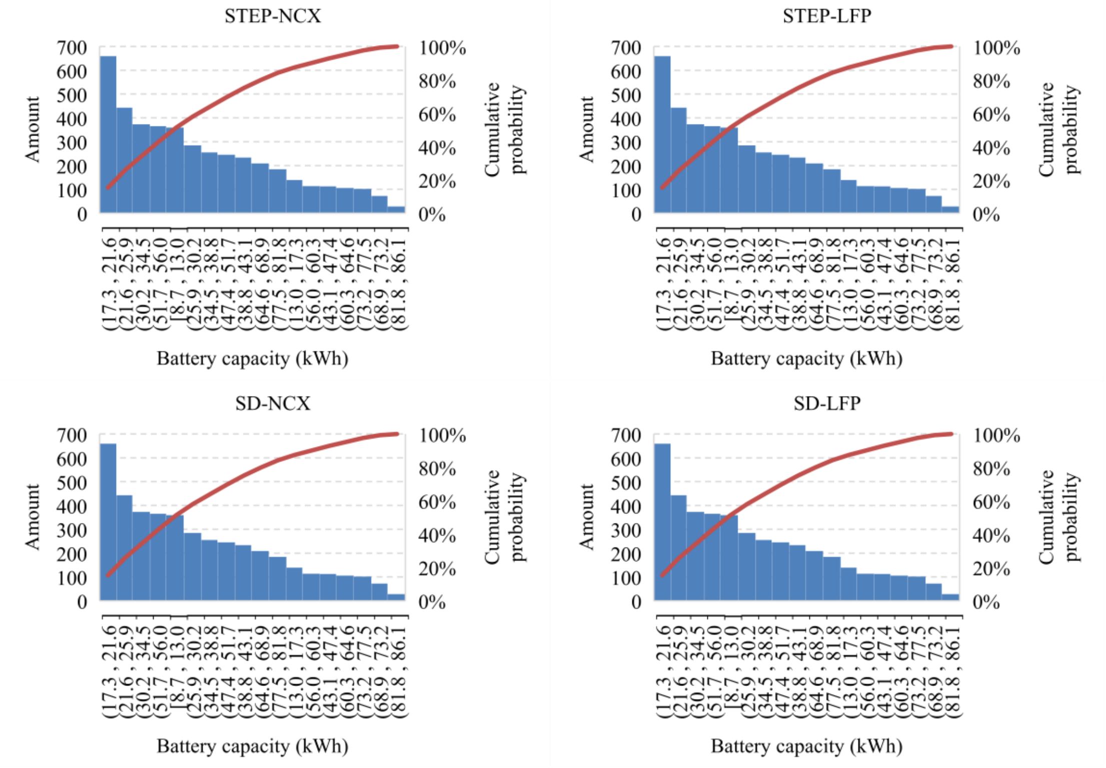

Supplementary Fig. 11: Battery capacity distribution for India battery stock by 2050.

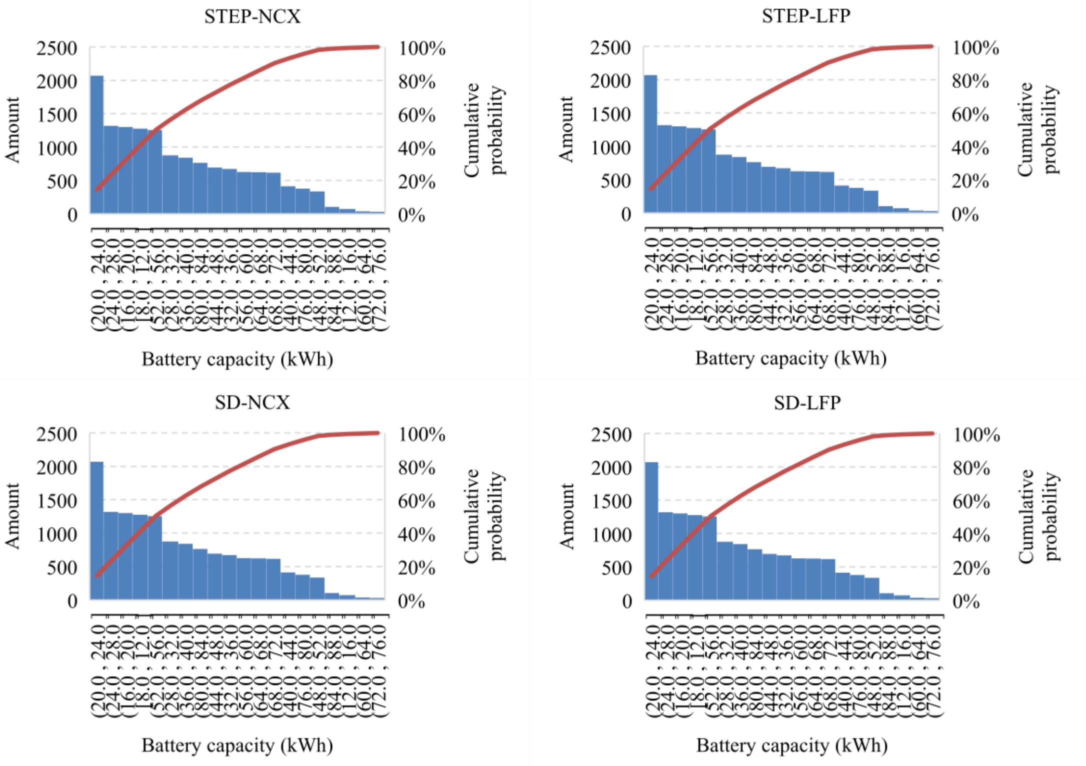

Supplementary Fig. 12: Battery capacity distribution for EU battery stock by 2050.

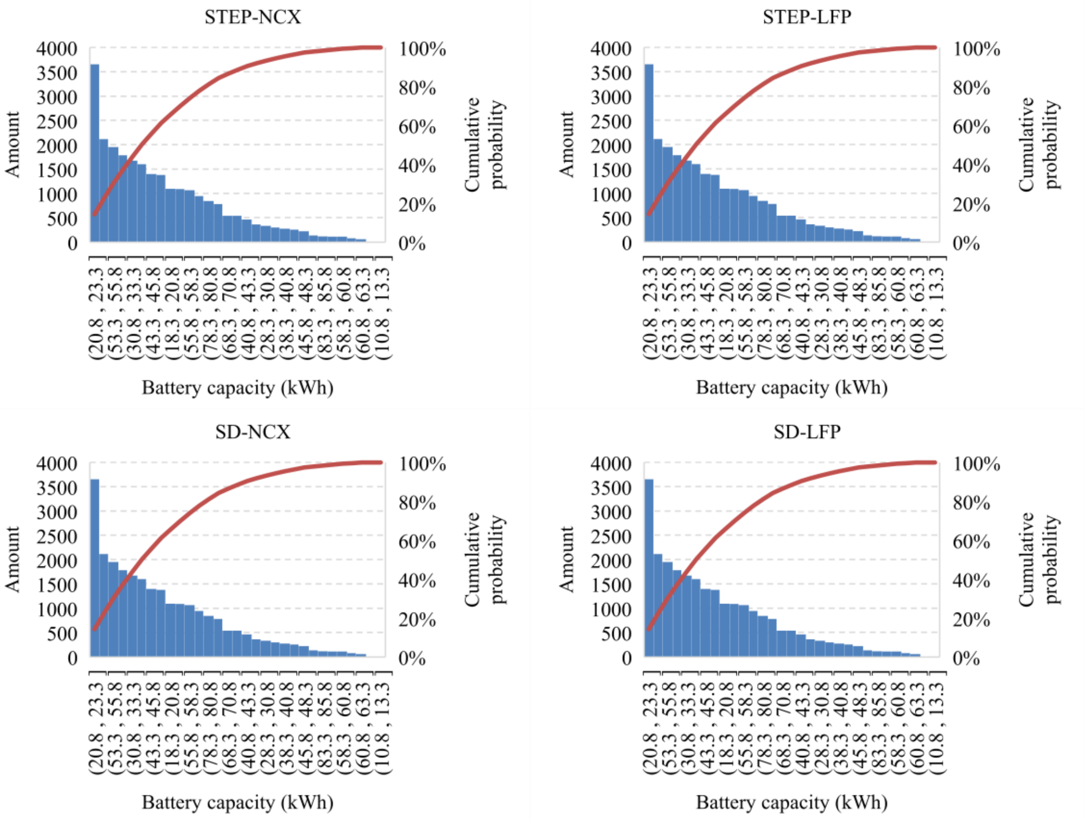

Supplementary Fig. 13: Battery capacity distribution for US battery stock by 2050.

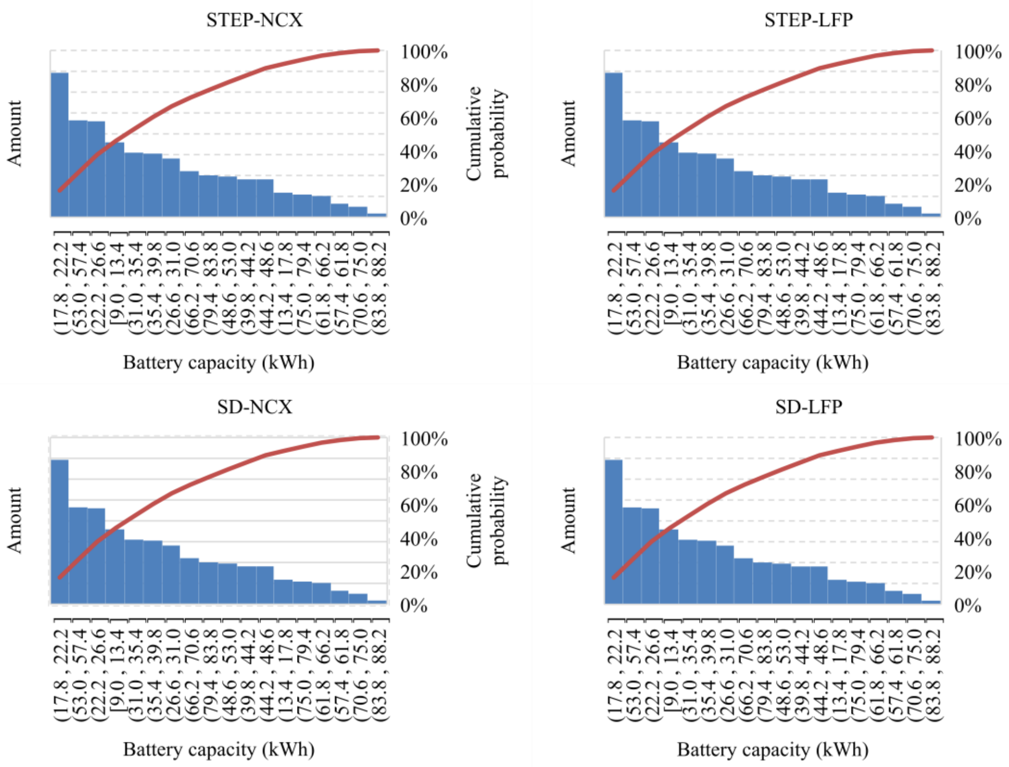

Supplementary Fig. 14: Battery capacity distribution for RoW battery stock by 2050.

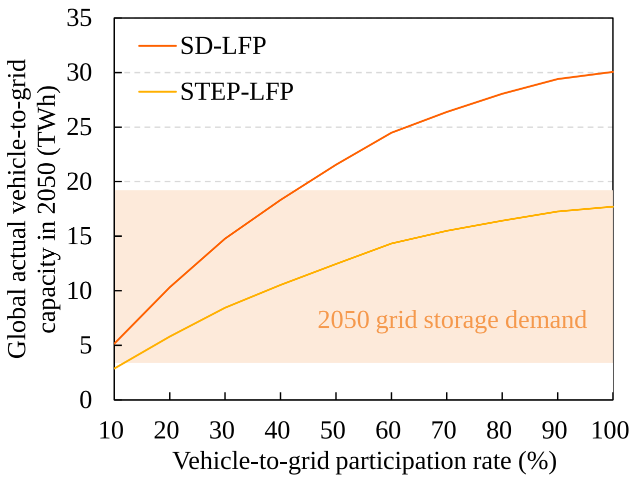

Supplementary Fig. 15: Global actual vehicle-to-grid capacity as a function of participation rates in STEP-LFP and SD-LFP scenarios, and comparison to grid storage capacity demand in 2050.

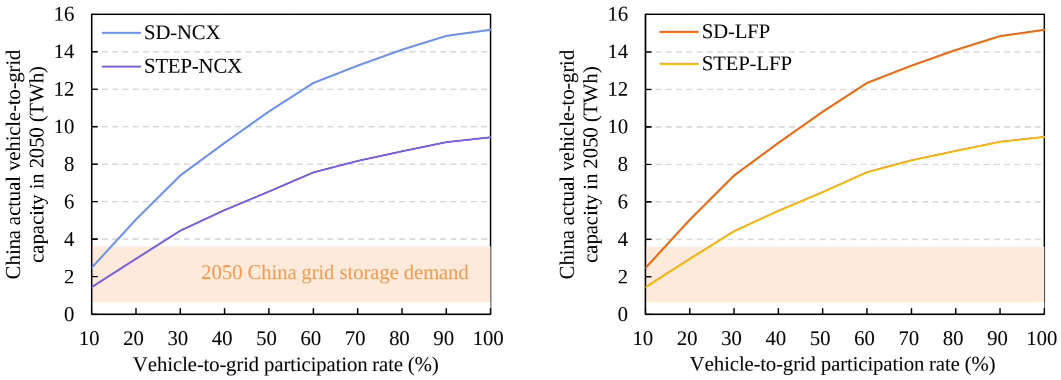

Supplementary Fig. 16: China actual vehicle-to-grid capacity as a function of participation rate and comparison to grid storage capacity demand in 2050.

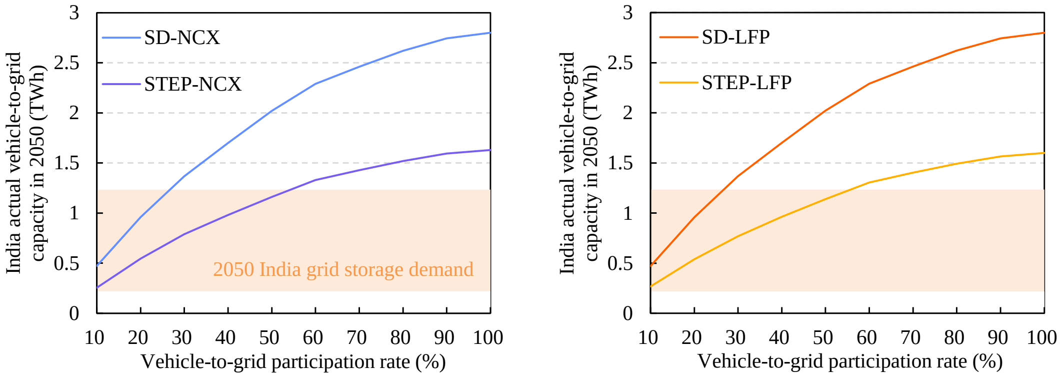

Supplementary Fig. 17: India actual vehicle-to-grid capacity as a function of participation rate and comparison to grid storage capacity demand in 2050.

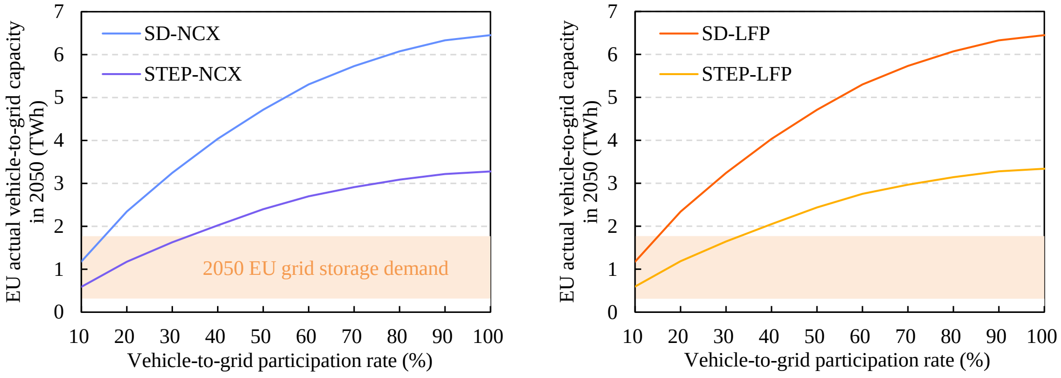

Supplementary Fig. 18: EU actual vehicle-to-grid capacity as a function of participation rate and comparison to grid storage capacity demand in 2050.

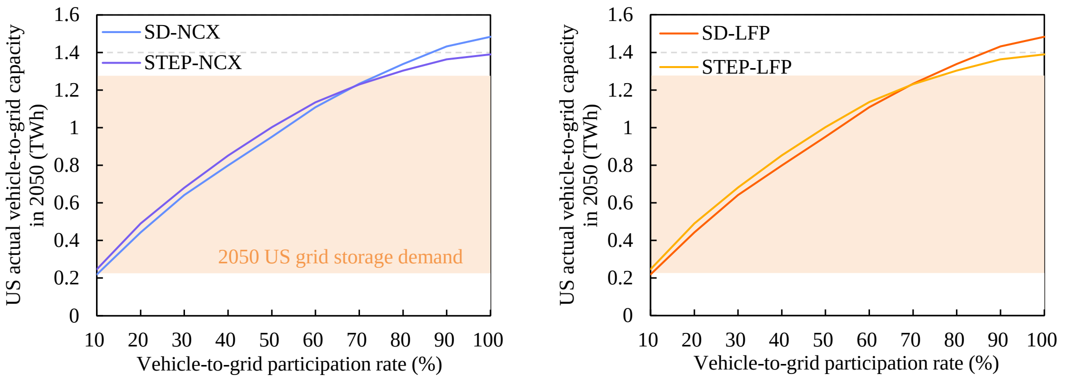

Supplementary Fig. 19: US actual vehicle-to-grid capacity as a function of participation rate and comparison to grid storage capacity demand in 2050.

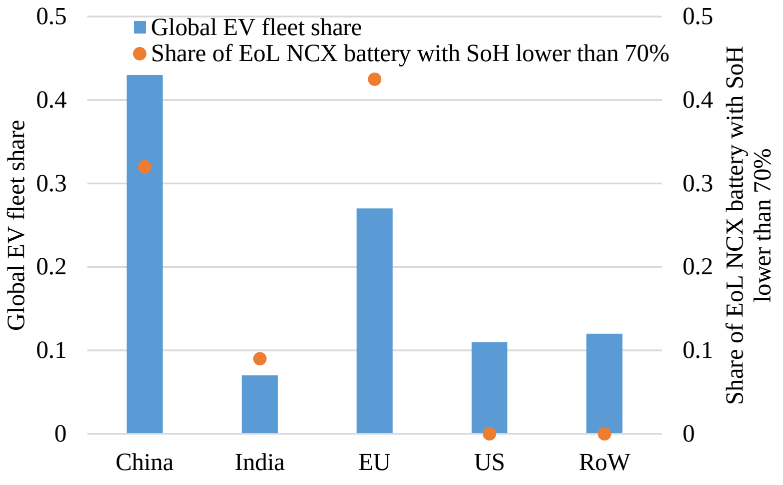

Supplementary Fig. 20: Global share of retired NCX batteries with SoH lower than 70% in total retired NCX batteries (i.e., repurposing rate per year). Repurposing rate per year = number of collected batteries with relative SoH above 70% per year / number of collected batteries per year.

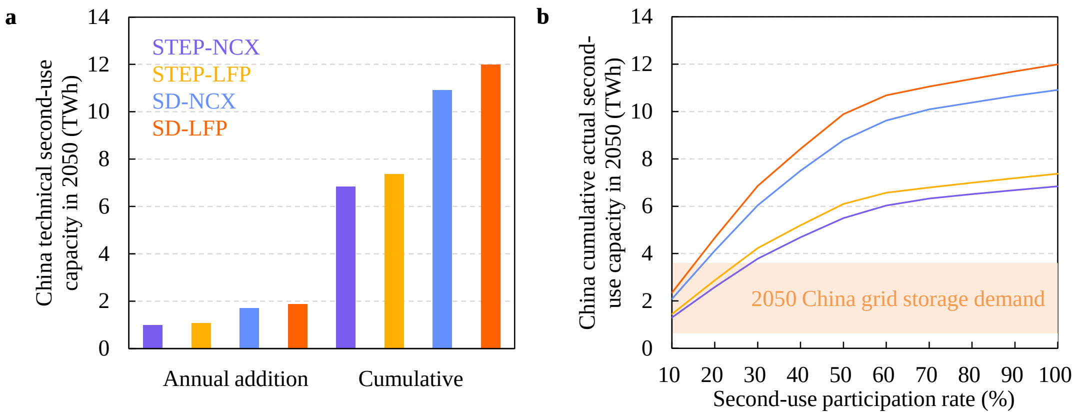

Supplementary Fig. 21: China available second-use capacity in 2050. **a** Annual addition and cumulative technical capacity in 2050. Capacity refers to the technically available capacity

considering battery degradation, or maximum theoretical potential second-use capacity without considering the battery second-use participation rate. **b** Impacts of second-use participation rate on cumulative actual second-use capacity and a comparison to storage demand in 2050 (orange shading).

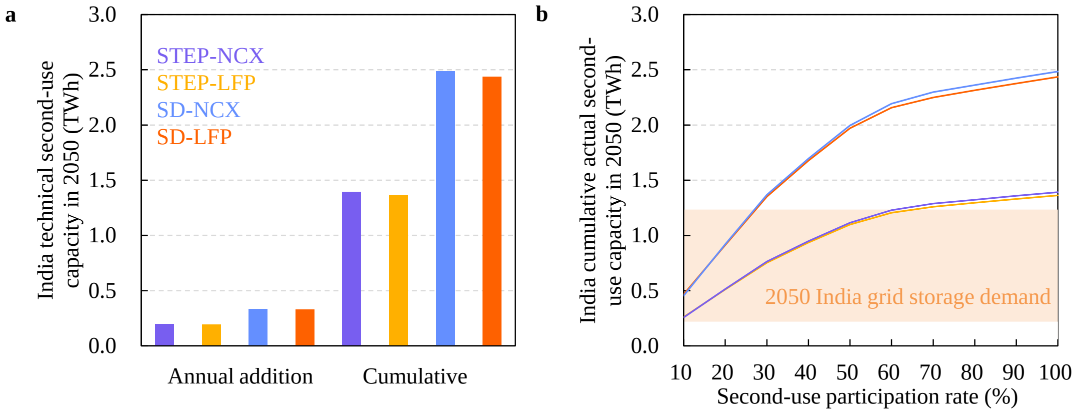

Supplementary Fig. 22: India available second-use capacity in 2050. a Annual addition and cumulative technical capacity in 2050. Capacity refers to the technically available capacity considering battery degradation, or maximum theoretical potential second-use capacity without considering the battery second-use participation rate. **b** Impacts of second-use participation rate on cumulative actual second-use capacity and a comparison to storage demand in 2050 (orange shading).

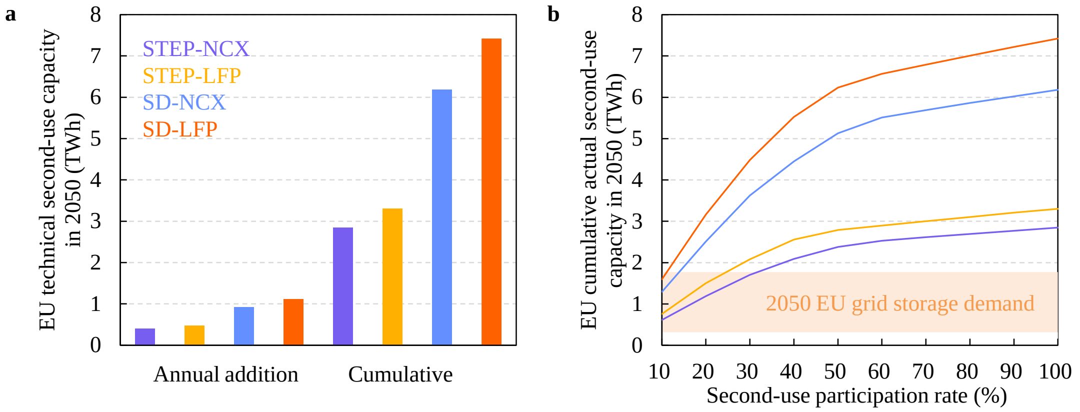

Supplementary Fig. 23: EU available second-use capacity in 2050. a Annual addition and cumulative technical capacity in 2050. Capacity refers to the technically available capacity considering battery degradation, or maximum theoretical potential second-use capacity without considering the battery second-use participation rate. **b** Impacts of second-use participation rate on cumulative actual second-use capacity and a comparison to storage demand in 2050 (orange shading).

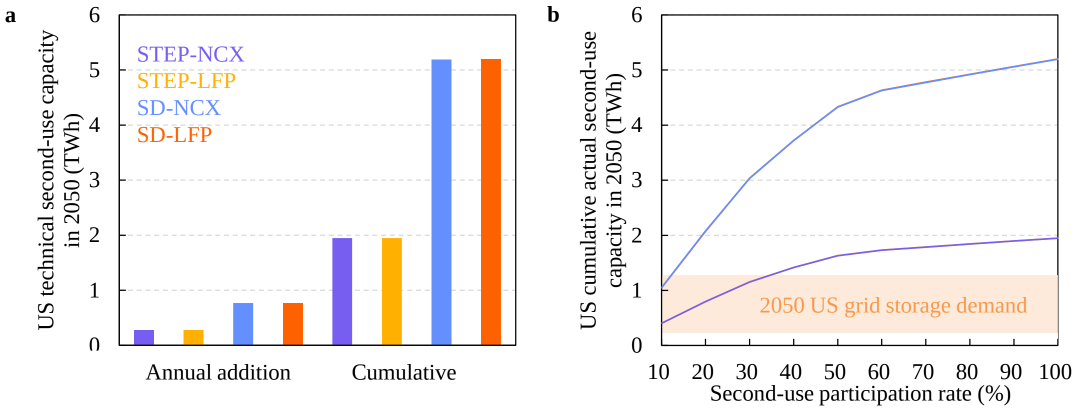

Supplementary Fig. 24: US available second-use capacity in 2050. a Annual addition and cumulative technical capacity in 2050. Capacity refers to the technically available capacity considering battery degradation, or maximum theoretical potential second-use capacity without considering the battery second-use participation rate. **b** Impacts of second-use participation rate on cumulative actual second-use capacity and a comparison to storage demand in 2050 (orange shading).

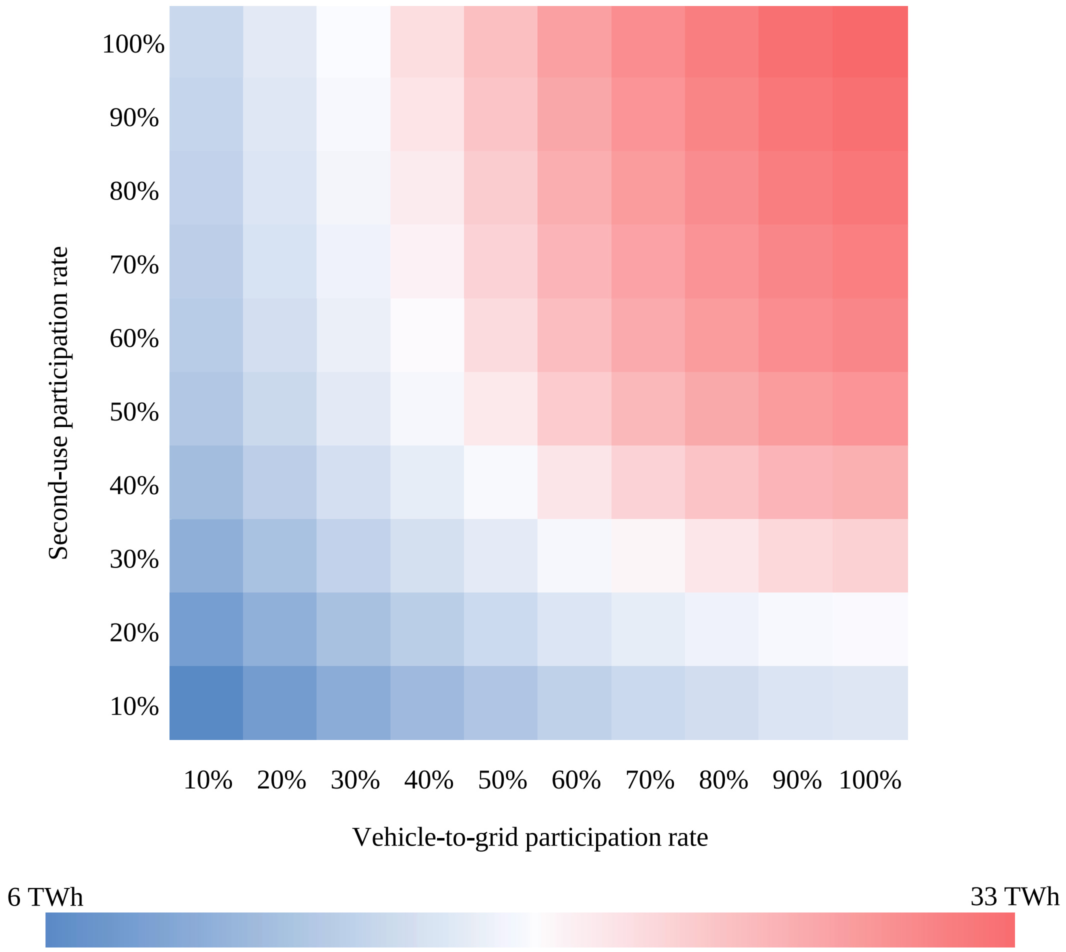

Supplementary Fig. 25: Total actual available capacity under various conditions in STEP-LFP scenario in 2050. Blue, white, and red colors depict minimum, average, and maximum values.

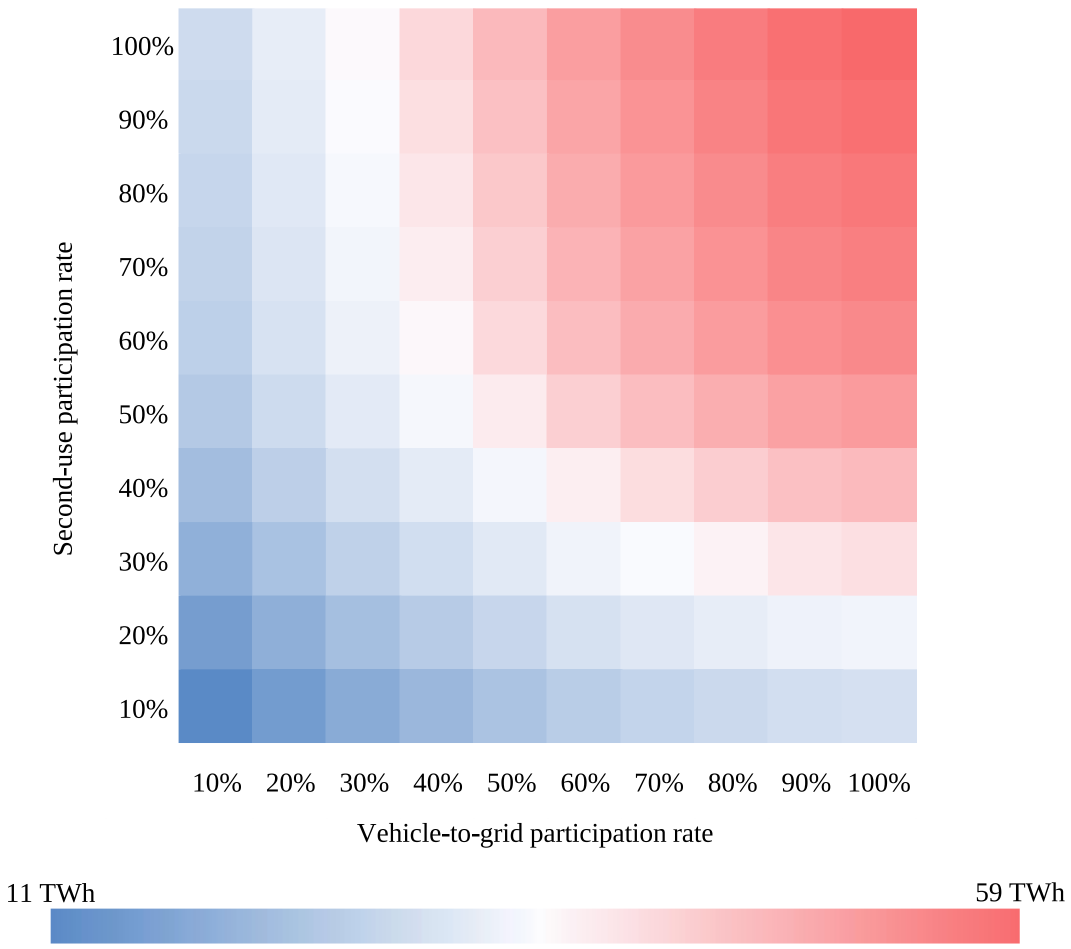

Supplementary Fig. 26: Total actual available capacity under various conditions in SD-NCX scenario in 2050. Blue, white, and red colors depict minimum, average, and maximum values.

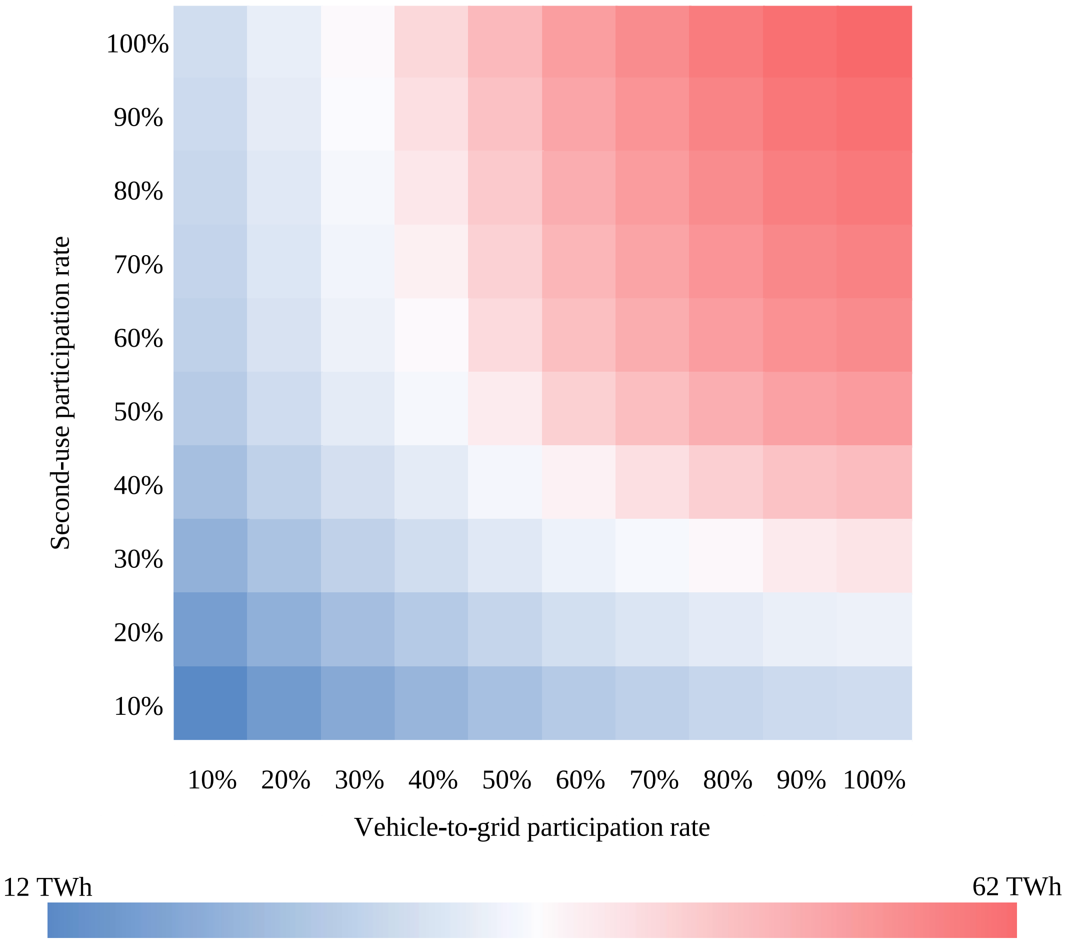

Supplementary Fig. 27: Total actual available capacity under various conditions in SD-LFP scenario in 2050. Blue, white, and red colors depict minimum, average, and maximum values.

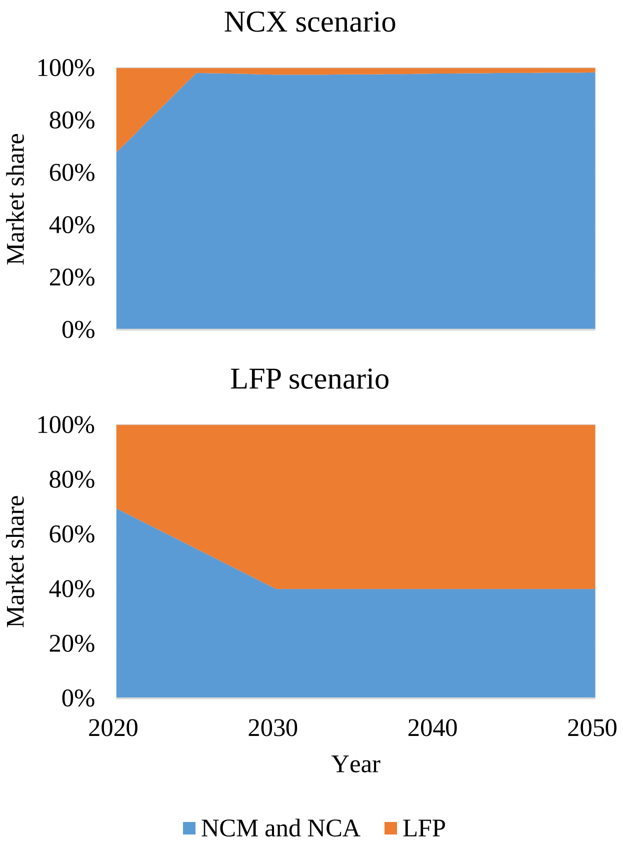

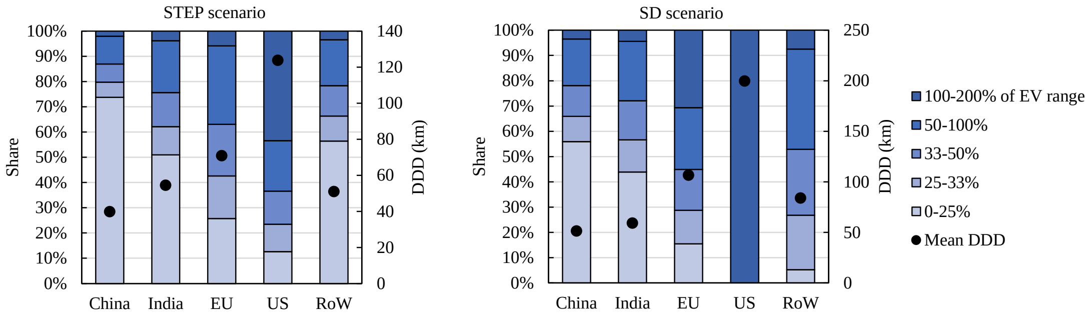

Supplementary Fig. 28: Daily driving distance (DDD) distributions for small BEV across counties/regions. The historic DDD distribution for EU is collected from Spritmonitor.de[4]. Combined with the IEA's projection of future EV fleet energy consumption for China, India, EU, US, and RoW[5], we compile the future DDD distributions for countries/regions.

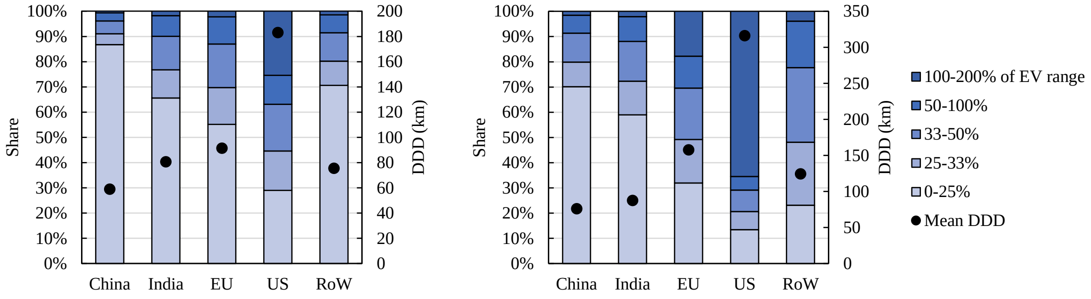

Supplementary Fig. 29: Daily driving distance (DDD) distributions for mid-size BEV across counties/regions. The historic DDD distribution for EU is collected from Spritmonitor.de[4]. Combined with the IEA's projection of future EV fleet energy consumption for China, India, EU, US, and RoW[5], we compile the future DDD distributions for countries/regions.

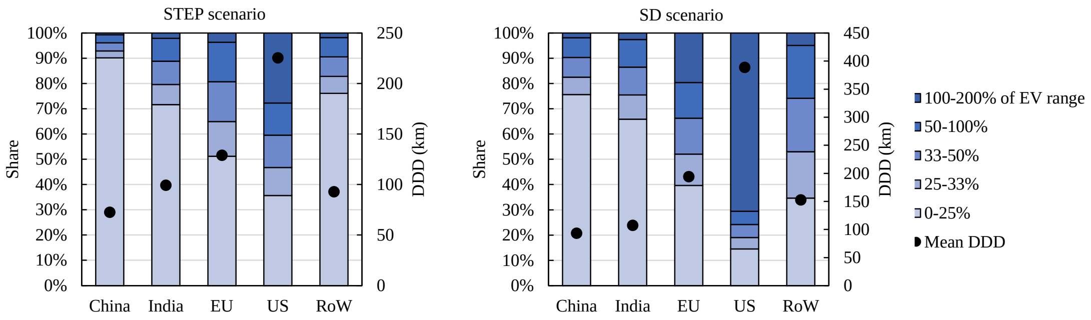

Supplementary Fig. 30: Daily driving distance (DDD) distributions for large BEV across counties/regions. The historic DDD distribution for EU is collected from Spritmonitor.de[4]. Combined with the IEA's projection of future EV fleet energy consumption for China, India, EU, US, and RoW[5], we compile the future DDD distributions for countries/regions.

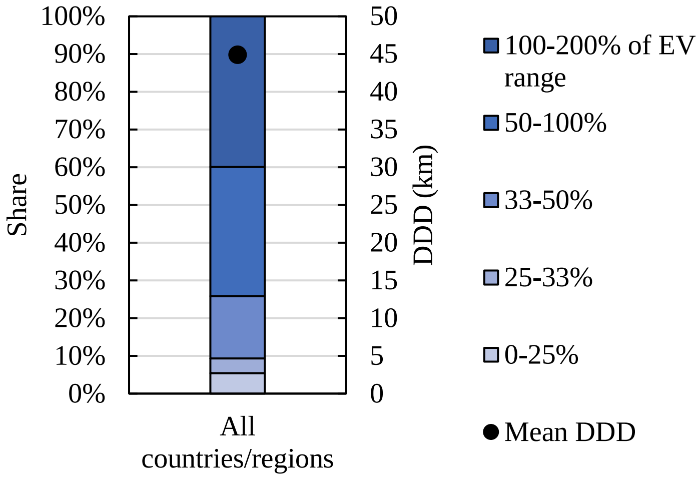

Supplementary Fig. 31: Supplementary Fig. 8: Daily driving distance (DDD) distributions assumed for PHEVs for all counties/regions.

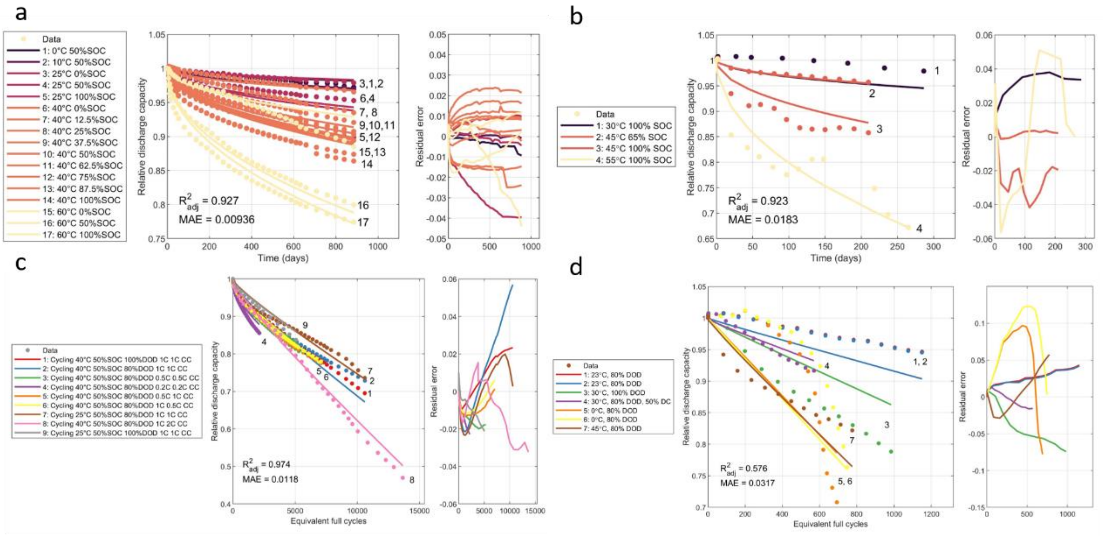

Supplementary Fig. 32: Battery degradation model fitting results. a calendar life aging of LFP**. b** calendar life aging of NCM. **c** cycling life aging of LFP. **d** cycling life aging of NCM. Residual errors are plotted to the right of each fit.

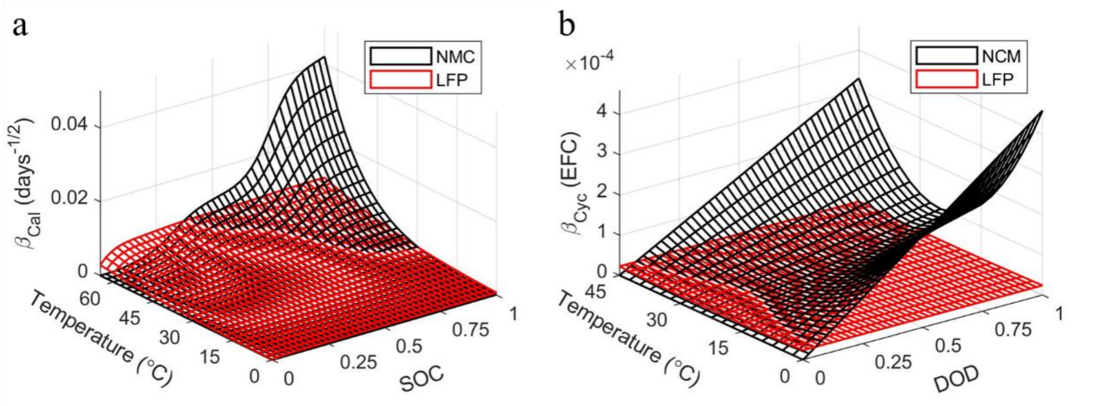

Supplementary Fig. 33: LFP and NCM battery degradation rates. a Calendar life degradation rate versus the square-root of time as a function of temperature and SoC (state-of-charge). **b** Cycle life degradation rate versus energy throughput, in units of EFCs (equivalent full cycles), as a function of temperature and DOD (depth-of-discharge).

Supplementary Table

Supplementary Table 1: Future grid storage capacity demand. IEA = International Energy Agency. IRENA = International Renewable Energy Agency. BNEF = Bloomberg New Energy Finance. SD scenario = sustainable development scenario. Remap = Renewable Energy Roadmap. PES = Planned Energy Scenario. The "Planned Energy Scenario (PES)" is the primary reference case for this study, providing a perspective on energy system developments based on governments' current energy plans and other planned targets and policies (as of 2019), including Nationally Determined Contributions under the Paris Agreement unless the country has more recent climate and energy targets or plans. TES = Transforming Energy Scenario. The "Transforming Energy Scenario (TES)" describes an ambitious, yet realistic, energy transformation pathway based largely on renewable energy sources and steadily improved energy efficiency (though not limited exclusively to these technologies). This would set the energy system on the path needed to keep the rise in global temperatures to well below 2 degree Celsius (°C) and towards 1.5°C during this century. Unit: TWh. TWh = 10^9 kWh.

Reference	Capacity demand	Scenarios	2030	2040	2050	Annual growth rate /increasing factor in 2030~2050	Annual growth rate /increasing factor in 2040~2050
IEA[6]	Stationary storage batteries	SD	/	2.9884	/		
IRENA[7]	Behind the meter storage batteries	Remap	/	/	9		
IRENA[8]	Electricity storage energy capacity	Reference scenario	7.22	/	/		
IRENA[8]	Electricity storage energy capacity	Doubling scenario	13.58	/	/		
IRENA[9]	Stationary storage	PES	0.37	/	3.4	0.12/9.19	
IRENA[9]	Stationary storage	TES	0.745	/	9	0.13/12.08	
BNEF[10]	Energy storage	/	/	2.85	/		

	installations						
Storage Lab[2]	Flexibility grid storage capacity	Optimistic approaches	/	2.8	8.8		0.12/3.14
Storage Lab[2]	Flexibility grid storage capacity	Conservative approaches	/	8.8	19.2		0.08/2.18

Supplementary Table 2: Selected EV models for modeling daily driving distance (DDD) distributions and driving cycles.

Vehicle type and class	EV models for modeling DDD distribution	Representative model for modeling drive cycles
Small BEV	Smart fortwo, Mitsubishi i-MiEV, BMW i3, Volkswagen e-Golf	Mitsubishi i-MiEV
Mid-size BEV	Nissan Leaf, Mercedes-Benz B250e, Honda Clarity EV, Hyundai Ioniq Electric, Tesla Model 3	Nissan Leaf 30 kWh
Large BEV	Tesla Model S, Kia Soul Electric, Hyundai Kona Electric	TESLA Model S60 2WD
PHEV	Toyota Prius, Ford C-MAX Energi Plug-In Hybrid, Hyundai Ioniq Plug-in Hybrid	Prius Prime

Supplementary Table 3: Optimized parameters for LFP and NCM degradation model.

Parameter	LFP	NCM
k_{Cal}	1.9234E-3 (days$^{0.5}$)	4.0149E-4 (days$^{0.5}$)
E_a	3.0233E4 (J/mol·K)	5.9178E4 (J/mol·K)
α	-0.05590	-1
k_{Cyc}	2.93583E-6	4.3131332E-6
A	1.4761E-11	0.3549361
B	7.4008E-3	1.2308964E-4
C	0.082035	0
D	0.0313111	1
G	0.33344256	0.6149392
H	331.652158	63.619859

Supplementary Table 4: Sensitivity analysis of battery capacity per vehicle on total actual available capacity in 2050. In this sensitivity analysis, we assume all BEVs are small BEVs equipped with a battery with a capacity of 33 kWh.

			Vehicle-to-grid participation rate									
			**10%**	**20%**	**30%**	**40%**	**50%**	**60%**	**70%**	**80%**	**90%**	**100%**
**STEP-NCX**	**Second-use participation rate**	**10%**	8.1	9.5	10.7	11.7	12.5	13.4	13.9	14.3	14.7	14.9
		**20%**	7.9	9.3	10.5	11.5	12.4	13.2	13.7	14.2	14.6	14.8
		**30%**	7.8	9.1	10.4	11.3	12.2	13.0	13.6	14.0	14.4	14.6
		**40%**	7.6	8.9	10.2	11.1	12.0	12.8	13.4	13.8	14.2	14.4
		**50%**	7.3	8.7	9.9	10.9	11.8	12.6	13.1	13.6	13.9	14.1
		**60%**	6.9	8.2	9.5	10.4	11.3	12.2	12.7	13.1	13.5	13.7
		**70%**	6.2	7.5	8.7	9.7	10.6	11.4	11.9	12.4	12.8	13.0
		**80%**	5.2	6.6	7.8	8.8	9.7	10.5	11.0	11.5	11.8	12.0
		**90%**	4.0	5.4	6.6	7.6	8.4	9.3	9.8	10.2	10.6	10.8
		**100%**	2.7	4.0	5.3	6.2	7.1	7.9	8.5	8.9	9.3	9.5
**STEP_LFP**	**Second-use participation rate**	**10%**	8.6	9.9	11.1	12.1	13.0	13.9	14.4	14.8	15.2	15.4
		**20%**	8.4	9.7	11.0	11.9	12.8	13.7	14.2	14.6	15.0	15.2
		**30%**	8.2	9.5	10.8	11.7	12.6	13.5	14.0	14.4	14.8	15.0
		**40%**	8.0	9.3	10.5	11.5	12.4	13.3	13.8	14.2	14.6	14.8
		**50%**	7.8	9.1	10.3	11.3	12.2	13.0	13.6	14.0	14.4	14.6
		**60%**	7.4	8.7	9.9	10.9	11.8	12.6	13.2	13.6	14.0	14.2
		**70%**	6.6	8.0	9.2	10.1	11.0	11.9	12.4	12.9	13.2	13.4
		**80%**	5.6	7.0	8.2	9.1	10.0	10.9	11.4	11.9	12.3	12.5
		**90%**	4.3	5.7	6.9	7.8	8.7	9.6	10.1	10.6	10.9	11.1
		**100%**	2.8	4.2	5.4	6.4	7.2	8.1	8.6	9.1	9.5	9.7
**SD-NCX**	**Second-use participation rate**	**10%**	15.8	18.2	20.3	21.9	23.4	24.7	25.6	26.4	27.0	27.3
		**20%**	15.5	17.9	19.9	21.6	23.0	24.4	25.3	26.0	26.7	27.0
		**30%**	15.1	17.5	19.6	21.2	22.7	24.0	24.9	25.7	26.3	26.6
		**40%**	14.8	17.2	19.2	20.8	22.3	23.7	24.5	25.3	25.9	26.2
		**50%**	14.3	16.7	18.7	20.4	21.8	23.2	24.1	24.8	25.5	25.8
		**60%**	13.4	15.8	17.8	19.5	21.0	22.3	23.2	24.0	24.6	24.9
		**70%**	11.9	14.3	16.3	17.9	19.4	20.8	21.7	22.4	23.1	23.4
		**80%**	10.1	12.5	14.5	16.1	17.6	19.0	19.9	20.6	21.3	21.6
		**90%**	7.7	10.0	12.1	13.7	15.2	16.6	17.4	18.2	18.8	19.1
		**100%**	5.1	7.4	9.5	11.1	12.6	14.0	14.8	15.6	16.2	16.5
**SD-LFP**	**Second-use participation rate**	**10%**	16.9	19.3	21.3	22.9	24.4	25.8	26.7	27.4	28.0	28.3
		**20%**	16.5	18.9	20.9	22.6	24.0	25.4	26.3	27.0	27.7	28.0
		**30%**	16.1	18.5	20.5	22.2	23.7	25.0	25.9	26.6	27.3	27.6
		**40%**	15.7	18.1	20.1	21.8	23.2	24.6	25.5	26.2	26.9	27.2
		**50%**	15.3	17.6	19.7	21.3	22.8	24.2	25.0	25.8	26.4	26.7
		**60%**	14.4	16.8	18.9	20.5	22.0	23.3	24.2	25.0	25.6	25.9
		**70%**	12.8	15.2	17.2	18.9	20.4	21.7	22.6	23.3	24.0	24.3
		**80%**	10.9	13.2	15.3	16.9	18.4	19.8	20.6	21.4	22.0	22.3
		**90%**	8.2	10.6	12.6	14.3	15.8	17.1	18.0	18.8	19.4	19.7
		**100%**	5.3	7.7	9.8	11.4	12.9	14.2	15.1	15.9	16.5	16.8

Supplementary Notes

Supplementary Note 1

As shown in Supplementary Fig. 4, we compile the trip driving cycle based on a standard US combined driving cycle (i.e., 55% UDDS city driving and 45% HWY highway driving). We first model the required trip distance and time for UDDS city driving and HWY highway driving, respectively. By comparing the required driving distance with the distance of the standard driving cycle, the required multiples (i.e., the repeated times of standard UDDS or HWY driving cycle) and downsizing factor (the downscaling of standard UDDS or HWY driving cycle to satisfy a small driving distance) are modeled, respectively, thus scaling up or down of standard driving cycle to the required driving distance. Supplementary Fig. 5 shows the driving cycle example of mid-size BEV, where the mean driving distance between 33%-50% EV range is 126.3 km. A 63.1 km of trip distance requires 2 multiples of standard UDDS city driving and 1 multiple of standard HWY highway driving, as well as 1 downsized standard UDDS driving distance with a downsizing factor of 1.11 and 1 downsized standard HWY driving with a downsizing factor of 1.38.

Supplementary Note 2

According to degradation models fit with aging data from state-of-the-art NCM and LFP batteries, LFP batteries show lower levels and less variance of degradation than NCX as LFP is less sensitive to temperature variation, state-of-charge, and depth of discharge in both calendar-life and cycle-life degradation rates (Supplementary Figs. 32 and 33). For a mid-size battery electric vehicle (BEV), an increase of daily driving distance (DDD) from 0%-25% EV range to 100%-200% of EV range could reduce the relative battery State-of-health (SoH) at 8 years (i.e., battery lifetime warranty by most EV manufacturers) by 5.5-22% for NCM and 1-1.5% for LFP, depending on temperature conditions (see Supplementary Data for degradation for different EV size and type). Higher utilization of plug-in hybrid vehicle (PHEV) batteries leads to higher degradation for PHEV batteries than for BEV batteries. Battery degradation variations among countries/regions are driven by driving intensity and climate conditions; the lifetime of NCM batteries in Europe is expected to be substantially shorter than other regions due to increased degradation caused by cycling at low average temperatures, while the lifetime of LFP batteries is shortest in India due to increased calendar degradation rate at high average temperatures (see Supplementary Figs. 28~31 for DDD distributions, Supplementary Data for city temperature and battery degradation).

Glossary

Dynamic battery stock model
EV: electric vehicles
BEV: battery electric vehicle
PHEV: plug-in hybrid electric vehicle
LFP: lithium-iron-phosphate / graphite battery
NCM: lithium Nickel Cobalt Manganese Oxide / graphite battery
NCA: lithium Nickel Cobalt Aluminum Oxide / graphite battery
NCX: NCM and NCA, with X denoting manganese or aluminum

EV use model
Ambient temperature: the temperature of the air surrounding the EVs under consideration
Daily driven distance (DDD): assumed as the mean value of DDD distribution
State of Charge (SoC): level of charge of a battery relative to its rated capacity, and the units of SoC are percentage points (0% = empty; 100% = full)
C_{rate}: the charge or discharge current divided by the battery's capacity to store an electrical charge. The unit of the **C_{rate}** is hour^{-1}
Depth of discharge (DOD): the fraction or percentage of the battery's capacity which is currently removed from the battery with regard to its (fully) charged state
Equivalent full cycles (EFCs): the charge throughput of partial cycles relative to a full charge/discharge cycle

Battery degradation model
Rated capacity: the maximum energy of the battery at the start of life
Battery degradation: the amount of charge a rechargeable battery can deliver at the rated voltage decreases with use, depending on lots of stress factors: Ambient temperature SoC, Crate, DoD, and EFCs
Battery capacity: a property of that a battery's maximum capability to store the energy at a given moment in time and conditions, as the battery degradation
Relative SoH: state of health, is assumed as Battery capacity / Rated capacity

Vehicle-to-grid model
Theoretical battery stock capacity: on-board EV battery capacity of total EV fleet, without considering capacity lost due to battery degradation. Theoretical battery stock capacity = Rated capacity per EV * number of total EVs
Technical vehicle-to-grid capacity: availability of theoretical battery stock capacity for vehicle-to-grid applications, considering driving demand, battery degradation, and PHEV. Technical vehicle-to-grid capacity = Theoretical battery stock capacity – Battery capacity reserved for BEV driving – Battery capacity of PHEV - Battery capacity lost due to battery degradation
Vehicle-to-grid participation rate: Number of EVs participating in vehicle-to-grid /

Number of total EVs

Actual vehicle-to-grid capacity: availability of technical vehicle-to-grid capacity for vehicle-to-grid applications. Actual vehicle-to-grid capacity = number of EVs participating in vehicle-to-grid * technical vehicle-to-grid capacity per EV

Second-use model

Retired battery: battery out of service from first life of EV

Capacity per retired battery: battery capacity when coming to the end of the first life of EV

Collection rate per year: number of collected batteries per year / number of retired batteries per year. Number of collected batteries per year = number of repurposed batteries + number of recycled batteries

Repurposing battery: retired battery that is suitable for electricity storage. The model assumes collected battery with relative SoH above 70% will be repurposed

Recycled battery: retired battery that is collected for material recycling

Repurposing rate per year: rate of repurposing batteries in collected batteries. Repurposing rate per year = number of collected batteries with relative SoH above 70% per year / number of collected batteries per year

Recycling rate per year: rate of recycled batteries in collected batteries. Recycling rate per year = 1- repurposing rate per year

Technical second-use capacity per year: battery capacity of repurposed batteries per year. Technical second-use capacity per year = number of retired batteries per year * collection rate per year * repurposing rate per year * capacity per retired battery

Second-use utilisation rate per year: number of batteries utilised in second-use / number of repurposing batteries (or collected batteries with relative SoH above 70%) per year

Actual second-use capacity per year: availability of technical second-use capacity per year for second-use applications. Actual second-use capacity per year = technical second-use capacity per year * second-use utilisation rate per year * capacity per retired battery

References

1. Xu, C. et al. Future material demand for automotive lithium-based batteries. *Commun. Mater.* **1**, 99 (2020).
2. *Electric Insights Quarterly* (Drax, 2019). https://www.drax.com/wp-content/uploads/2019/12/191202_Drax_Q3_Report.pdf
3. *Global EV Outlook 2020* (IEA, 2020). https://www.iea.org/reports/global-ev-outlook-2020
4. *MPG and Cost Calculator and Tracker* (Spritmonitor, 2020). https://www.spritmonitor.de/en/
5. *Global EV Data Explorer* (IEA, 2021). https://www.iea.org/articles/global-ev-data-explorer
6. *Innovation in Batteries and Electricity Storage* (IEA, 2020). https://www.iea.org/reports/innovation-in-batteries-and-electricity-storage
7. *Global energy transformation: A roadmap to 2050 (2019 edition)* (IRENA, 2019). https://www.irena.org/publications/2019/Apr/Global-energy-transformation-A-roadmap-to-2050-2019Edition
8. *Electricity storage and renewables: Costs and markets to 2030* (IRENA, 2017). https://www.irena.org/publications/2017/oct/electricity-storage-and-renewables-costs-and-markets
9. *Global Renewables Outlook: Energy transformation 2050* (International Renewable Energy Agency, 2020). https://www.irena.org/publications/2020/Apr/Global-Renewables-Outlook-2020
10. *Energy Storage Investments Boom As Battery Costs Halve in the Next Decade* (BloombergNEF, 2019). https://about.bnef.com/blog/energy-storage-investments-boom-battery-costs-halve-next-decade/
