# Supplementary material for: Electric vehicle batteries alone could satisfy short-term grid storage demand by as early as 2030
Source: Nat Commun. 2023 Jan 17;14:119. doi: 10.1038/s41467-022-35393-0 (PMC9845221; doi:10.1038/s41467-022-35393-0)
Supplement: Supplementary file 3 — Description of Additional Supplementary Files [file 41467_2022_35393_MOESM3_ESM.pdf]

**Description of Additional Supplementary Files**

File Name: Supplementary Data 1
Description: EV fleet scenario, battery chemistry scenario, and battery degradation estimations used for assessing energy storage potential of EV batteries.
